# Emerging treatment modalities for systemic therapy in hepatocellular carcinoma

**DOI:** 10.1186/s40364-021-00319-3

**Published:** 2021-08-21

**Authors:** Xin Qing, Wenjing Xu, Jingjing Zong, Xuanlong Du, Hao Peng, Yewei Zhang

**Affiliations:** Department of General Surgery, Zhongda Hospital, Medical School, Southeast University, Nanjing, 210009 China

**Keywords:** Hepatocellular carcinoma, Targeted therapy, Immunotherapy, Tyrosine kinase inhibitors, Immune checkpoint inhibitors, Signalling pathway

## Abstract

Hepatocellular carcinoma (HCC) has long been a major global clinical problem as one of the most common malignant tumours with a high rate of recurrence and mortality. Although potentially curative therapies are available for the early and intermediate stages, the treatment of patients with advanced HCC remains to be resolved. Fortunately, the past few years have shown the emergence of successful systemic therapies to treat HCC. At the molecular level, HCC is a heterogeneous disease, and current research on the molecular characteristics of HCC has revealed numerous therapeutic targets. Targeted agents based on signalling molecules have been successfully supported in clinical trials, and molecular targeted therapy has already become a milestone for disease management in patients with HCC. Immunotherapy, a viable approach for the treatment of HCC, recognizes the antigens expressed by the tumour and treats the tumour using the immune system of the host, making it both selective and specific. In addition, the pipeline for HCC is evolving towards combination therapies with promising clinical outcomes. More drugs designed to focus on specific pathways and immune checkpoints are being developed in the clinic. It has been demonstrated that some drugs can improve the prognosis of patients with HCC in first- or second-line settings, and these drugs have been approved by the Food and Drug Administration or are nearing approval. This review describes targeting pathways and systemic treatment strategies in HCC and summarizes effective targeted and immune-based drugs for patients with HCC and the problems encountered.

## Introduction

Liver cancer is the fourth most common cause of cancer deaths worldwide [1]. Hepatocellular carcinoma (HCC) is a prominent and complex subtype of primary liver cancer, accounting for over 90% of cases [2]. Hepatectomy, liver transplantation, and ablation are potentially curative for patients diagnosed with early-stage HCC [3, 4]; however, conventional systemic chemotherapy loses its survival benefits for advanced patients [5]. It is absolutely essential to develop unique treatment modalities to better manage HCC.

Recently, there has been significant progress in the understanding of signalling pathways, techniques to detect tumour progression, and drugs to block pathway activity, providing opportunities to develop precise treatments [6]. Multiple aberrant pathways in HCC have been characterized such as the Ras-Raf-MAPK, PI3K-AKT-mTOR, Wnt-β-catenin, and JAK-STAT pathways [7–10]. Drugs targeting these pathways have demonstrated encouraging survival benefits and bring considerable hope to patients with HCC [11]. Furthermore, immunotherapy has been suggested as a neopotential treatment for HCC patients [12]. Immune checkpoint inhibitors (ICIs) have been used successfully in patients with HCC [13]. These targeted and immune-based agents, including sorafenib, lenvatinib, regorafenib, and nivolumab are already available as clinical options for first-line or second-line treatment [14–16].

With the development of molecular medicine, systemic therapy significantly enhances quality of life and has become a major means of treatment for HCC, particularly for combination therapy and subsequent therapy [17, 18]. However, targeted therapies are often correlated with considerable resistance and adverse events (AEs), presenting a substantial challenge to further broadening existing treatment approaches for patients with HCC [19].

This review describes important pathways in HCC and discusses recent developments in targeted therapy for these tumours, including the evolution of therapeutic modalities and available options, with the goal of specifying precise treatment protocols for HCC in future studies.

### Targeting pathways in HCC

**Fig. 1:**
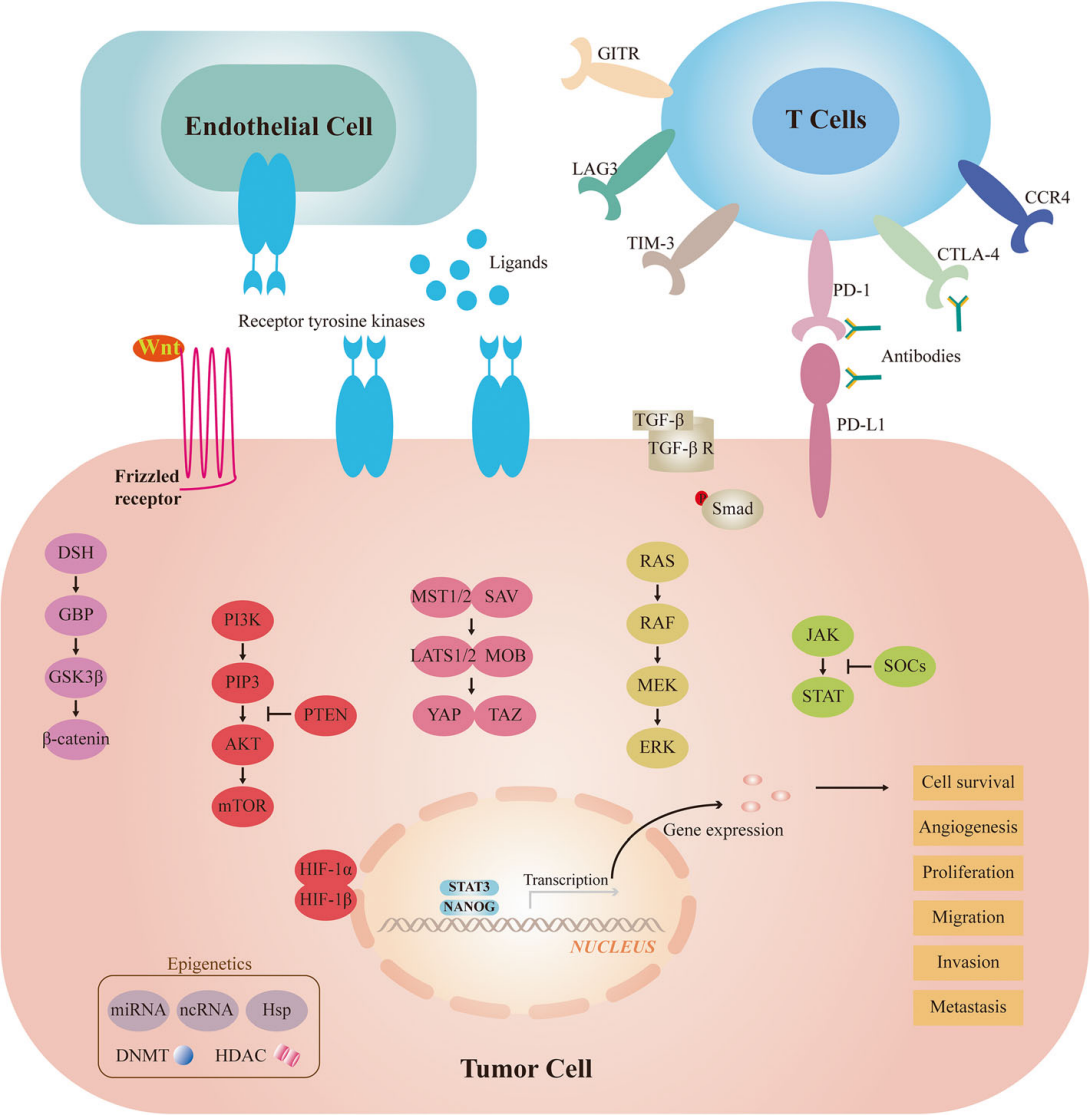
Signaling pathways and therapeutic targets in focus for HCC

Hepatocarcinogenesis is a multielement and multistep process and is associated with aberrant activation of diverse signalling pathways (Fig. 1) involving both receptor and nonreceptor actions of tyrosine kinase proteins [20]. The exploration of tumour signal transduction has been an active area in the field of basic tumour research and is the theoretical basis for various molecular targeted drugs.

### Ras-Raf-MAPK pathway

The Ras-Raf-MAPK pathway is a classic cellular signalling pathway that is generally associated with cell growth and proliferation and is located downstream of several tyrosine kinase receptors [21]. In HCC, the overexpression of several molecules in this pathway such as ras, raf-1, and ERK has been previously reported [22–24]. These variations often indicate a poor prognosis and short survival time in patients [25]. In addition, increased expression of the MAPK pathway is found in high-grade tumours [26]. Therefore, MAPK pathways may play a central role in liver carcinogenesis. This conclusion has been confirmed in preclinical trials, where specific MEK inhibitors blocked tumour angiogenesis and induced apoptosis by inhibiting this pathway [27]. Targeted drugs for precise molecules in this pathway have also shown promising antitumour activity in clinical trials [28, 29]. Additionally, the HBV X protein and HCV core protein can also activate the Ras-Raf-MAPK pathway [30, 31], and these studies indicate that this pathway may play a potential role in the progression of hepatitis to HCC.

### PI3K-AKT-mTOR pathways

The PI3K-AKT-mTOR pathway plays a vital role in regulating leading cellular processes such as cell survival and metabolism, and thus its deregulation is associated with malignant transformation and recurrence, including in liver cancer [32]. The analysis of tumour tissues from HCC patients revealed that mTOR signalling activity was observed in almost 50% of HCC samples [33]. This unrestricted activity is associated with abnormal PTEN as a tumour suppressor [34]. Notably, mutations in the PI3K-AKT-mTOR pathway are uncommon in HCC, and activation of this pathway is often largely due to ligand-dependent receptor activation [35]. When compared with normal samples, tumours containing aberrant levels of pathway components such as p-AKT, RICTOR, and pRPS6 are usually of a higher grade and have a worse overall prognosis [36, 37]. Moreover, cholangiocarcinoma, the second most prevalent primary liver cancer, also shows an upregulation of this pathway [38]. Some studies have demonstrated that a further increase in mTOR activation was identified in advanced HCC and in larger tumours [39]. Aberrant mTOR activation involves the PI3K-AKT and MAPK-ERK signalling pathways, which are often coinduced in HCC [40]. It has been reported that the blockade of both pathways boosts the response of HCC cells to sorafenib [41]. In addition, the PI3K-AKT-mTOR pathway is the cornerstone of the runaway signalling network in HCC and is a predominant driver in sorafenib resistance [42]. mTOR inhibitors are beneficial for active HCC patients after liver transplantation [43]. Various drugs targeting this pathway have shown excellent antitumour effects, particularly in combination with MAPK pathway inhibitors.

### Wnt-β-catenin pathways

Activation of the Wnt-β-catenin pathway in HCC is strongly related to early development, poorly differentiated tumours, earlier recurrence, and an adverse prognosis of HCC [44, 45]. Wnt functions as a regulator in liver regeneration and the self-renewal of progenitor and pluripotent stem cells and may be an optimal target for precise therapy [46]. Forty percent of HCC patients exhibit altered Wnt-β-catenin signalling, and nearly 20% of these mutations are caused by the β-catenin gene itself [47, 48]. The tissue analysis of HCC samples has demonstrated that the activation of β-catenin participates in abnormal cellular proliferation, tumour metastasis, and vascular invasion [49]. Hepatitis B and C virus infections induce overexpression of β-catenin, thus promoting hepatocarcinogenesis [50, 51].

### JAK-STAT pathways

In both normal and tumour cells, the JAK-STAT pathway serves multiple essential biological functions. Abnormalities of JAK-STAT can be found in over 45% of liver cancers [52]. In HCC, aberrantly activated JAK-STAT signalling results in the malfunction of downstream target genes, thereby controlling survival, cell division, angiogenesis, and metastasis [53]. The activation of STATs in tumours is considerably higher than that in adjacent liver tissues, and this degree of activation is responsible for the poor prognosis of tumour patients [54]. The JAK-STAT pathway is also responsible for the preservation of cancer stem cells with tumour propagation capacity in HCC and the establishment of an immunosuppressive microenvironment [55]. Given the oncogenic activity of JAK-STAT activation, particularly in the absence of a STAT3 disorder, targeting this pathway appears to be a favourable strategy for treating HCC.

### Hippo-YAP pathways

The Hippo-YAP pathway has critical roles in controlling organ size and tissue homeostasis. However, deregulation of Hippo-YAP signalling has been demonstrated in multiple cancers and leads to various oncogenic effects [56]. Signalling is activated not only through the interaction between appropriate extracellular ligands and their cellular receptors but also via cell polarity and adhesion [57]. The expression levels of the YAP protein and mRNA were very different in normal and cancerous liver tissues, and excessive activation of YAP in mice led to hepatocellular carcinogenesis [58]. Elevated YAP levels are an early event in the development of HCC and are largely attributed to gene amplification and posttranscriptional regulation [59]. Notably, nearly 50% of HCC patients display altered YAP overexpression and nuclear localization [60], and detection of the components of Hippo-YAP signalling may provide prognostic value for patients with HCC, as the amount of nuclear YAP is highly correlated with the survival time of patients [61].

### Proangiogenic pathways

HCC is a highly vascularized tumour, and angiogenesis is responsible for its tumorigenesis. Proangiogenic factors such as VEGF, FGF, PDGF, and hepatocyte growth factor trigger endothelial cell tyrosine kinases and downstream intracellular signalling via Ras-Raf-mTOR-Wnt pathways to activate angiogenesis [62]. These factors are expressed on endothelial cells and stimulate tumour neoangiogenesis, invasion, and migration. VEGF receptor expression is upregulated in HCC specimens, and elevated levels are negatively correlated with the overall survival (OS) of patients [63]. FGF is overexpressed in HCC compared with normal tissues, and this variation is recognized as oncogenic activity [64]. Dysregulation of PDGF resulting in tumorigenesis has been identified in several tumours, including HCC, and PDGF expression is markedly increased in highly metastatic cancer [65]. EGFR and IGFR are similar tyrosinase receptors, except that they have less impact on angiogenesis.

### Treatment advances in systemic therapy

**Fig. 2:**
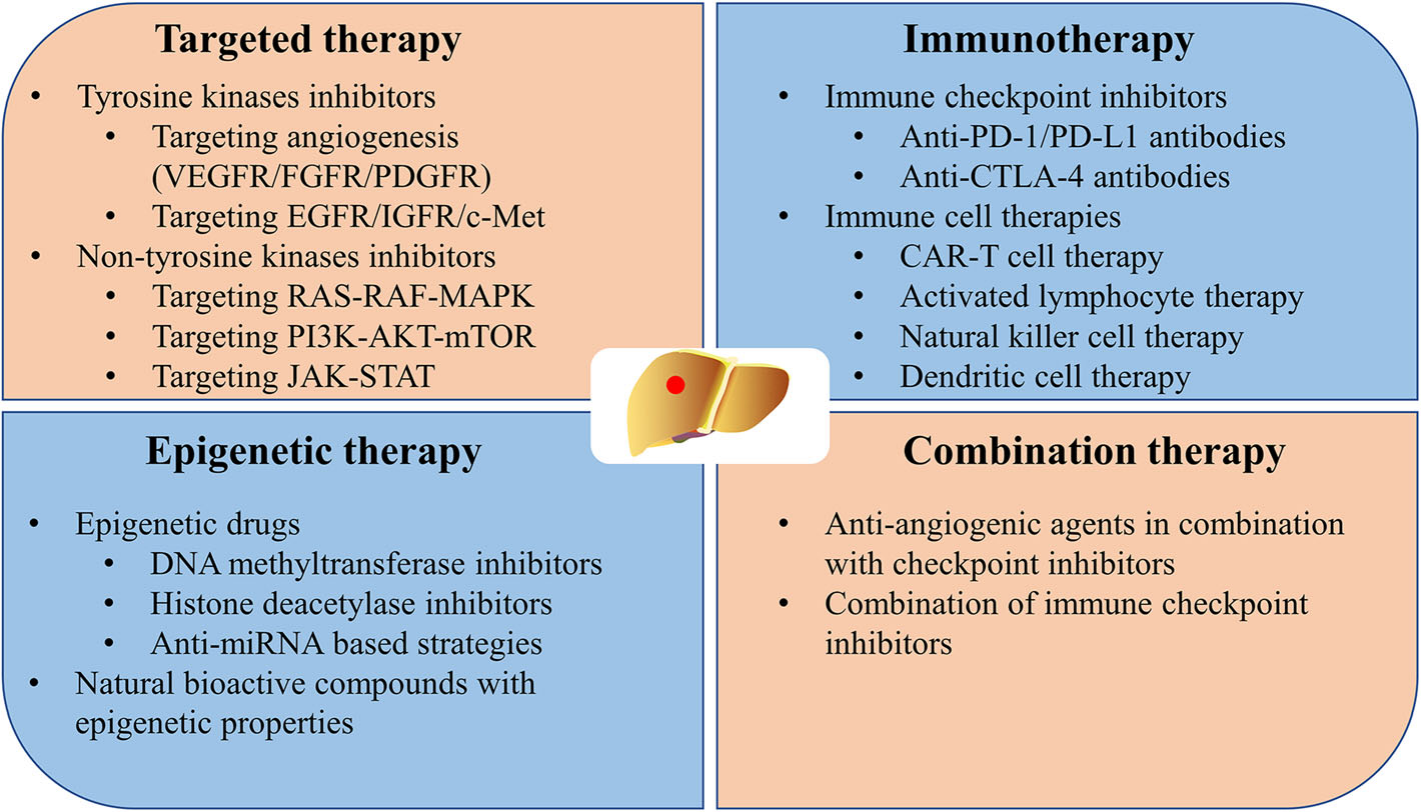
Systemic treatment strategies in HCC

The occurrence of HCC is related to the complex interaction between signal transduction pathways, the tumour microenvironment, and the genetic background, which leads to many opportunities for precise treatment. The treatment regimens for HCC have undergone a major transformation with the investigation of the molecular mechanism, and many new treatment approaches have become available as therapeutic options (Fig. 2). Tyrosine kinase inhibitors (TKIs) and ICIs have improved the quality of life and greatly extended the survival time of patients with HCC. Table 1 provides a list of clinical trials evaluating systemic therapeutic agents in HCC.

**Table 1:** Clinical trials of systemic therapy in HCC

Therapy	Time	Trial	Line	Drug	Control	Primary outcomes (HR; 95% CI)
Targeted therapy						
	2005	NCT00033462	First	Erlotinib	None	6-month PFS rate: 32%
	2007	SHARP	First	Sorafenib	Placebo	OS: 10.7 vs. 7.9 (0.69; 0.55–0.87)
	2008	Asia-Pacific	First	Sorafenib	Placebo	OS: 6.5 vs. 4.2 (0.68; 0.50–0.93)
	2011	NCT00699374	First	Sunitinib	Sorafenib	OS: 7.9 vs. 10.2 (1.3; 1.13–1.5)
	2011	NCT00604721	First	Selumetinib	None	TTP: 2
	2012	BRISK-FL	First	Brivanib	Sorafenib	OS: 9.5 vs. 9.9 (1.06; 0.93–1.22)
	2012	BRISK-PS	Second	Brivanib	Placebo	OS: 9.4 vs. 8.2 (0.89; 0.69–1.15)
	2013	NCT00427973	First	Cediranib	None	3-month PFS rate: 77%
	2014	EVOLVE-1	Second	Everolimus	Placebo	OS: 7.6 vs. 7.3 (1.05; 0.86–1.27)
	2015	NCT01210495	Second	Axitinib	None	16-week DCR rate: 42.3%
	2015	REACH	Second	Ramucirumab	Placebo	OS: 9.2 vs. 7.6 (0.87; 0.72–1.05)
	2016	NCT01232296	First	Dovitinib	Sorafenib	OS: 8.0 vs. 8.4 (1.27; 0.90–1.79)
	2017	RESORCE	Second	Regorafenib	Placebo	OS: 10.6 vs. 7.8 (0.63; 0.50–0.79)
	2018	REFLECT	First	Lenvatinib	Sorafenib	OS: 13.6 vs. 12.3 (0.95; 0.79–1.06)
	2018	NCT01004003	First	Nintedanib	Sorafenib	TTP: 5.5 vs. 4.6 (1.44; 0.81–2.57)
	2018	NCT01915589	First	Refametinib	None	ORR: 0%; DCR: 56.3%
	2018	METIV-HCC	Second	Tivantinib	Placebo	OS: 8.4 vs. 9.1 (0.97; 0.75–1.25)
	2019	CELESTIAL	Second	Cabozantinib	Placebo	OS: 10.2 vs. 8.0 (0.76; 0.63–0.92)
	2019	REACH-2	Second	Ramucirumab	Placebo	OS: 8.5 vs. 7.3 (0.71; 0.531–0.949)
	2020	NCT02645981	First	Donafenib	Sorafenib	OS: 12.1 vs. 10.3 (0.831; 0.699–0.988)
	2020	NCT02329860	Second	Apatinib	Placebo	OS: 8.7 vs. 6.8 (0.785; NA)
Immunotherapy						
	2013	NCT01008358	First	Tremelimumab	None	PR: 17.6%; DCR: 76.4%
	2017	Checkmate-040	Second	Nivolumab	None	ORR: ~ 17%
	2018	KEYNOTE-224	Second	Pembrolizumab	None	ORR: 17%
	2019	Checkmate-459	First	Nivolumab	Sorafenib	OS: 16.4 vs. 14.7 (0.85; 0.72–1.02)
	2019	KEYNOTE-240	Second	Pembrolizumab	Placebo	PFS: 3.0 vs. 2.8 (0.718; 0.570–0.904)
	2020	NCT02989922	Second	Camrelizumab	None	ORR: 14.7%

OS, overall survival; PFS, progression-free survival; DCR: disease control rate; TTP: time to progression; PR: partial response; ORR: object response rate; HR, hazard ratio; CI, confidence interval

* Primary outcomes (months or rate)

### Targeted therapy

Within the multitude of molecules known to be growth promoters, members of the family of protein tyrosine kinases have a central role in regulating tumour-initiating activity [66], and this is the main object of targeted therapy.

Since the availability of sorafenib as an antiangiogenic and antiproliferative agent in 2007, TKIs have revolutionized the management of HCC, ushering in the epoch of systemic therapies. Sorafenib prolonged the median overall survival (mOS) of patients with advanced HCC from 8 to 11 months with a tolerated toxicity profile [67]. In a subsequent trial, this endpoint was further extended to 14.7 months [68]. However, no additional valid systemic treatment alternatives have been identified for nearly a decade after the introduction of sorafenib. In recent years, several novel treatment compounds have demonstrated benefits as first- and second-line regimens. New eligible agents, such as lenvatinib in the first-line setting and regorafenib, cabozantinib, and ramucirumab in the second-line setting, have already been demonstrated to improve clinical outcomes, although the mOS remains at approximately one year [69, 70]. With all of these drugs, the study design was similar to that of sorafenib, which typically selected patients with advanced HCC in stage B or C BCLC and assessed OS as the primary endpoint (Table 1) [14, 71]. To date, TKIs are effective treatments that have received widespread support for the treatment of advanced HCC worldwide.

Interestingly, the drugs showing significant clinical effects are almost all multikinase inhibitors (MKIs), which may be attributed to the multiple activation mechanism of hepatocellular carcinogenesis [72]. If patients with advanced HCC have failed locoregional treatment or cannot receive a liver transplant, MKIs are considered acceptable treatment agents. Certainly, if the goal is a driver mutation, the role of a single-target inhibitor may be primarily beneficial [73], but several single-target TKIs have been tested in HCC without positive results, including erlotinib, refametinib, and selumetinib [74–76].

Nevertheless, targeted therapies are correlated with AEs, modest efficacy, and regular occurrence of drug resistance. Fortunately, breakthroughs in immunotherapy have brought fresh hope to HCC patients.

### Immunotherapy

The object of immunotherapy is the immune system and tumour microenvironment of patients, rather than directly killing or interfering with tumour cells [77]. Common immune checkpoints in the tumour are PD-1/PD-L1 and CTLA-4. The association between PD-1 and HCC was first seen in patients with HBV infection, where higher PD-1 expression was associated with an increased viral load and a fold-increased risk of HCC [78]. There are various monoclonal antibodies approved by the FDA for use in the clinic: nivolumab, pembrolizumab, and camrelizumab for anti-PD-1 and atezolizumab, aveluma, and durvalumab for anti-PD-L1 [79]. Nivolumab is the first ICI approved as a second-line therapy by the FDA to treat advanced HCC based on results from the CheckMate-040 trial [80]. Subsequently, pembrolizumab and camrelizumab also received regulatory approval in advanced HCC patients treated with sorafenib [81, 82]. Accordingly, nivolumab and pembrolizumab may be particularly beneficial for patients who are not suitable for TKIs [83]. Although CTLA-4 inhibitors have been available for solid tumours, they have not been adequately demonstrated to improve survival for treating this malignancy [84]. Common anti-CTLA-4 antibodies such as ipilimumab and tremelimumab are currently used in combination with anti-PD/anti-PD-L1 in the treatment of liver cancer. Nevertheless, immunotherapy is only approved for a limited number of cancer patients for various reasons, and it is necessary to explore additional immune checkpoints beyond PD-1/PD-L1.

In some tumour patients, ICIs do not elicit tumour-specific T-cell activity and are less effective in treating patients. However, this effect can be improved by cell therapy [85]. Cell therapy, such as CAR-T, TCR-T or CAR-NK, is an emerging field of treating malignant tumours. Several cell therapies for HCC have already successfully entered clinical trials and have presented excellent experimental results [86–89]. Therefore, cell therapies can significantly prevent the recurrence of HCC and represent a powerful treatment approach for HCC.

With the improvement of immunotherapy and the positive effects of immunotherapeutic drugs in the treatment of HCC, interest in adjuvant immunotherapy after HCC resection has increased. Immunologic drugs such as ^131^I-metuximab significantly improved the survival outcomes of patients after hepatectomy [90]. The rate of pathologic complete response was 29% in a phase II study evaluating nivolumab with or without ipilimumab in patients with resectable HCC [91]. This encouraging result has yet to be confirmed in the ongoing phase III trial. Neoadjuvant immunotherapy with nivolumab plus ipilimumab has demonstrated antitumour activity in an early phase trial [92]. Cytokine-induced killer (CIK) cell immunotherapy for early HCC brings a median RFS benefit of 14 months but lacks survival advantages in advanced HCC patients as an adjuvant therapy, but this result can also be enhanced by targeting myeloid-derived suppressor cells [93, 94]. A similar encouraging adjuvant immunotherapy is HSP70 mRNA-transfected dendritic cell therapy [86].

Clinical trials involving more ICIs for HCC are under investigation, including trials targeting transforming growth factor-β (TGF-β), T cell immunoglobulin and mucin-containing protein 3 (TIM-3), V-domain Ig suppressor of T cell activation (VISTA), T cell Ig and immunoreceptor tyrosine-based inhibitory motif domain (TIGIT) and lymphocyte activation gene-3 (LAG-3).

### Epigenetic therapy

Sequential epigenetic alterations in regulatory genes can lead to oncogene activation and inactivation or the loss of oncogenes, resulting in tumorigenesis [95]. Major elements of epigenetic mechanisms such as ncRNAs, DNA methylation, and histone modifications change their expression or function in the background of liver fibrosis, cirrhosis, and HCC [96]. Epigenetic dysregulation represents an important function in the aetiology of HCC, which has recently been widely recognized [97]. Generally, epigenetic alterations are largely intrinsically reversible and can be seen as therapeutic targets to block the progression of HCC. Numerous drugs that specifically target epigenetic modifiers have been developed and proven successful against multiple tumour models, and several of these are in clinical trials [98, 99].

### Combination therapy

#### Anti-angiogenic agents in combination with checkpoint inhibitors

For patients with solid tumours, vascular abnormalities can enable the tumour to evade surveillance and attack by the immune system, and these abnormalities arise from elevated angiogenic factors such as VGEF and angiopoietin 2 (ANG2). Agents targeting these molecules can normalize tumour vasculature, which in turn enhances tissue perfusion and tumour infiltration by immune cells, thereby improving the response to immunotherapy [100]. Additionally, preclinical evidence demonstrates that immunotherapy can improve the immunomodulatory effects of antiangiogenic therapy, as has been well illustrated in the available trial data [101]. Multiple clinical trials taking this into account have achieved the desired success. The combination of atezolizumab and bevacizumab (anti-VEGF) is intended to change the treatment landscape, as it is the first treatment to show superiority in front-line therapy for advanced HCC in recent years. It now appears to have an overwhelming advantage over the FDA-approved first-line options of sorafenib and lenvatinib [102]. Although specific VEGF/VEGFR inhibitors may enable antitumour immunity, multikinase inhibitors remain nonnegligible agents. Lenvatinib combined with pembrolizumab is another combination therapy for advanced HCC, and early data shows good prospects [103]. Lenvatinib plus AK104 (PD-1/CTLA-4 bispecific antibody) as a first-line therapy for unresectable HCC has shown promising antitumour activity and an acceptable safety profile [104]. This study was presented at the ASCO 2021 meeting and presents a few new ideas. Camerzumab (SHR-1210) in combination with apatinib has shown promising antitumour activity for HCC in a phase II study [105]. ICIs will probably become the backbone of systemic combination therapy for patients with HCC, but needs further study. The synergistic effects of ICIs and antiangiogenic drugs are being investigated in clinical trials (Table 2). This combination therapy represents an evolution of the present treatment options, which are supported by a powerful preclinical rationale.

**Table 2:** Ongoing trials investigating the combination therapy of TKIs and ICIs

Targeted therapy	Checkpoint inhibitor	Phase	Patients No.	Clinical setting	Status	NCT numbers
Lenvatinib	Nivolumab	I	30	HCC without prior systemic therapy	Active, not recruiting	NCT03418922
Lenvatinib	Nivolumab	II	50	Multinodular, advanced HCC	Recruiting	NCT03841201
Lenvatinib	Pembrolizumab	I	104	HCC	Active, not recruiting	NCT03006926
Lenvatinib	Pembrolizumab	III	750	Advanced HCC	Active, not recruiting	NCT03713593
Regorafenib	Nivolumab	I/II	60	HCC progressing under sorafenib	Recruiting	NCT04170556
Regorafenib	Nivolumab	II	42	Unresectable HCC	Recruiting	NCT04310709
Regorafenib	Nivolumab	III	496	Intermediate-Stage HCC	Not yet recruiting	NCT04777851
Regorafenib	Pembrolizumab	I	57	Advanced liver cancer without prior systemic therapy	Active, not recruiting	NCT03347292
Regorafenib	Pembrolizumab	II	119	Advanced or metastatic HCC after ICIs	Recruiting	NCT04696055
Cabozantinib	Nivolumab	I	15	Locally advanced HCC after definitive resection	Active, not recruiting	NCT03299946
Cabozantinib	Nivolumab	I	18	Advanced Solid Tumors in Patients with HIV Infection	Recruiting	NCT04514484
Cabozantinib	Pembrolizumab	II	29	Liver cancer not eligible for local therapy	Recruiting	NCT04442581
Sorafenib/lenvatinib	Atezolizumab	III	554	Locally advanced or metastatic and/or unresectable HCC participants following prior HCC treatment with atezolizumab and bevacizumab combination	Not yet recruiting	NCT04770896
Bevacizumab	Nivolumab	II	60	Advanced HCC	Recruiting	NCT04393220
Bevacizumab	Atezolizumab	III	662	HCC with high risk of recurrence after surgical resection or ablation	Recruiting	NCT04102098
Bevacizumab	Atezolizumab	III	150	Unresectable HCC without prior systemic treatment	Recruiting	NCT04487067
Bevacizumab	Atezolizumab	III	480	Locally advanced or metastatic HCC without prior systemic treatment	Completed	NCT03434379

#### Combination of immune checkpoint inhibitors

Because tumour escape mechanisms involve the aberrant expression of different immune checkpoint molecules, the combination of multiple ICIs for tumour treatment may result in enhanced efficacy (Table 3) [106]. The inhibition of PD-1/PD-L1 can activate prospective tumour immunity only if there are enough CD8+ T cells in the tumour tissue. Moreover, anti-CTLA-4 antibodies can increase the abundance of active CD8+ T cells by inhibiting the B7-CTLA-4 pathway and then promoting the infiltration of active CD8+ T cells into tumour tissue, thus enhancing the antitumour effect [107]. This principle provides the rationale for ongoing clinical trials of the simultaneous blockade of multiple immune checkpoints. The CheckMate 040 trial tested the effects of nivolumab plus ipilimumab in the second-line setting for advanced HCC patients [108]. Nivolumab in combination with ipilimumab had acceptable safety, an optimistic object response rate (ORR), and a durable response in that randomized trial. Another phase I/II trial to evaluate the efficacy and safety of durvalumab plus tremelimumab in unresectable HCC has also been reported [109]. The results of the experiment are exciting, and the next phase of the study should prove worthwhile. Finally, a promising direction of development is to establish more relevant preclinical models of HCC to identify the most potent combination strategies between anti-PD-1/PD-L1 antibodies and alternative immunomodulatory agents for further clinical development. There are various ongoing combination therapies of different ICIs in HCC (Table 3).

**Table 3:** Ongoing trials investigating the combination therapy of ICIs

Checkpoint inhibitors	Phase	Patients (No.)	Clinical setting	Status	NCT numbers
Nivolumab + Ipilimumab	I/II	32	Child-Pugh A HCC	Recruiting	NCT03682276
Nivolumab + Ipilimumab	I	50	Unresectable HCC with Child Pugh Class A cirrhosis	Active, not recruiting	NCT03203304
Nivolumab + Ipilimumab	II	30	HCC with potential surgical resection	Active, not recruiting	NCT03222076
Nivolumab + Ipilimumab	III	650	Advanced HCC without prior systemic therapy	Recruiting	NCT04039607
Nivolumab + Ipilimumab	II	40	HCC with potential for curative surgical resection	Recruiting	NCT03510871
Nivolumab + Mogamulizumab	I	96	locally advanced or metastatic solid tumors	Completed	NCT02476123
Nivolumab + Relatlimab	I	20	Potentially resectable HCC	Not yet recruiting	NCT04658147
Pembrolizumab + Bavituximab	II	28	Locally advanced or metastatic HCC not amenable to locoregional therapy	Recruiting	NCT03519997
Durvalumab+ Tremelimumab	III	1504	Unresectable HCC without prior systemic therapy	Recruiting	NCT03298451
Durvalumab+ Tremelimumab	I	32	Unresectable, locally advanced liver cancer after radioembolization	Suspended	NCT04605731
Durvalumab+ Tremelimumab	II	433	Advanced HCC	Active, not recruiting	NCT02519348

Combination therapies are currently in diverse phases of clinical exploitation to optimize antitumour efficacy. The combination of locoregional therapy with immunotherapy and targeted therapy for advanced HCC plays a huge role in the clinic, particularly for advanced HCC [110, 111].

### Currently approved treatment options

Systemic therapy has been recognized as a substantial benefit for patients with HCC. Table 4 summarizes the systemic therapeutic agents for HCC that are approved by the FDA.

**Table 4:** Systemic therapies approved for HCC by FDA

Agent	Mechanism	FDA-approved indications
First-line		
Sorafenib	Targeting VEGFR, PDGFR, c-KIT, RET, and Ras/Raf/MEK/ERK	As monotherapy in patients with unresectable or metastasis HCC
Lenvatinib	Targeting VEGFR, PDGFR, FGFR, RET, and SCFR	As monotherapy in patients with unresectable HCC
Atezolizumab + Bevacizumab	Anti-PD-L1 antibody + Anti-VEGF antibody	For patients with unresectable or metastatic HCC who have not received prior systemic therapy
Second-line		
Regorafenib	Targeting VEGFR, PDGFR, BRAF, FGFR, KIT, and RET	As monotherapy in sorafenib-experienced patients
Cabozantinib	Targeting VEGFR2, c-MET, AXL, KIT, and RET	As monotherapy in sorafenib-experienced patients
Ramucirumab	Anti-VEGFR antibody	As monotherapy in sorafenib-experienced patients with AFP of 400 ng/ml or higher
Nivolumab	Anti-PD-1 antibody	As monotherapy in sorafenib-experienced patients
Pembrolizumab Nivolumab + Ipilimumab	Anti-PD-1 antibody Anti-PD-1 antibody + Anti-CTLA-4 antibody	As monotherapy in sorafenib-experienced patients As combination therapy in sorafenib-experienced patients

### First-line therapy

#### Sorafenib

Until 2017, sorafenib was the only potent treatment for patients who entered an advanced stage of HCC or failed other therapies. It is an oral MKI that blocks the activities of receptor tyrosine kinases (VEGFR-2/3, PDGFR-β, c-Kit, FLT-3, and RET), downstream pathway kinases (Ras/Raf/MAPK and JAK/STAT), and other targets (B-Raf and c- Raf) [112]. Sorafenib extends the mOS from 7.9 months to 10.7 months compared with the placebo arm [113]. The trial provides a standard study scheme for evaluating promising drugs. Similar benefits were identified in a parallel phase III trial in an Asian population, mostly with HBV-infected patients [114]. In later clinical trials, the OS of sorafenib-experienced patients appears to have increased; for example, the OS of patients in Asia increased from 6.5 months to 11 months and that of patients in the western region increased from 10.7 months to 15.1 months [115]. In these trials, the AEs were mostly similar and manageable, including hand-foot skin reactions, rash or desquamation, hypertension, diarrhoea, fatigue, and ascites.

The success of sorafenib to treat advanced HCC seems beneficial for earlier clinical stages, but it did not obtain the expected benefit in the placebo-controlled clinical trial [116, 117]. Although the widespread use of sorafenib increases the survival time of patients with HCC, its effective rate is low due to the formation of drug resistance [118]. Subsequently, several targeted drugs such as sunitinib, brivanib, erlotinib, linifanib, and lapatinib have been tested in a phase III trial over the past decade, but further breakthroughs were not made until the emergence of two other oral tyrosinase inhibitors: lenvatinib and regorafenib.

#### Lenvatinib

Lenvatinib is an oral MKI that blocks the behaviours of VEGFR1–3, FGFR1–4, PDGFR, RET, and KIT. In a phase III noninferiority randomized controlled trial involving 954 unresectable HCC patients worldwide, the role of lenvatinib was compared with sorafenib as a first-line therapy [119]. In this study, the mOS as the primary endpoint in the lenvatinib and sorafenib groups was 13.6 months and 12.3 months, respectively. With the tumour evaluation results conducted by mRECIST, lenvatinib had a statistically significant improvement in all secondary endpoints compared with sorafenib. Progression-free survival (PFS) and ORRs were significantly higher in the lenvatinib group (PFS: 7.4 months vs. 3.7 months; ORRs: 24.1% vs. 9.2%). Moreover, lenvatinib had greater survival benefits for HBV-related HCC patients [120]. Additionally, lenvatinib plays a promising role in delaying the deterioration of diseases, including role function, pain, and diarrhoea [121].

Notably, the treatment-related AEs were similar in patients receiving lenvatinib or sorafenib, although accompanied by a different incidence, predominantly of hypertension and thrombocytopenia. This may be because the median duration of the treatment was longer in the lenvatinib group than in the sorafenib group [119].

These trial results indicated that lenvatinib is not inferior to sorafenib as a first-line setting and is regarded as an optimal candidate for sorafenib. Consequently, lenvatinib as a first-line treatment was recently supported by the ESMO, EASL, and ASCO guidelines [122–124].

#### Atezolizumab plus bevacizumab

Recently, reliable data on the combination of atezolizumab and bevacizumab have been presented, and this therapeutic approach is approved by the FDA and is used in patients with unresectable or metastatic HCC who have not received prior systemic treatment [125]. The combination of angiogenesis inhibitors and PD-L1 inhibitors is designed to inhibit high levels of angiogenesis and overactivity of VEGF and PD-L1 in HCC tumours. In an initial study, atezolizumab plus bevacizumab was efficacious and clinically meaningful, and it had obvious clinical benefits compared with atezolizumab alone [126]. Furthermore, this combination therapy was compared with sorafenib in the phase III IMbrave150 trial. The OS rates at 12 months of the combination group and the sorafenib group were 67.2 and 54.6%, respectively. The combination treatment reduced the risk of death by 42% (HR: 0.58, *P* = 0.0006). The mPFS was 6.8 months and 4.3 months in the respective groups, and the risk of disease progression or death was reduced by 41% (HR: 0.59, *P* < 0.0001) [102]. OS and PFS, as the primary endpoints of the study, met the predefined statistical threshold. Atezolizumab plus bevacizumab compared with sorafenib delayed the deterioration of quality of life (time to deterioration: 11.2 months vs. 3.6 months, respectively). In addition, the grade 3–4 side effects of combination treatment were mainly hypertension and proteinuria, with no difference from those of atezolizumab and bevacizumab alone. In Chinese patients with advanced HCC, combination therapy has also resulted in substantial clinical improvements in OS and PFS [127].

Atezolizumab plus bevacizumab to treat HCC was first included in the ASCO guidelines in 2020 on the basis of the IMbrave150 trial [124].

#### Subsequent therapy

The efficacy of first-line agents in patients with HCC may be restricted by contraindications, drug resistance, and AEs, and it is necessary to replace first-line agents with sequential treatment in these situations.

#### Regorafenib

Following the successful inclusion of regorafenib in the second-line setting, sequential therapy has been recognized as the standard modality. Regorafenib has profound antiproliferative and antiangiogenic effects on tumour cells by inhibiting VEGFR signal transduction, thereby regulating the tumour microenvironment. Its chemical structure differs from sorafenib only by a fluorine-carbon atom on the central benzene ring [128]. A randomized controlled phase III trial indicated mOS and mPFS improvement in further treatment of advanced HCC in sorafenib-experienced patients (mOS: 10.6 months vs 7.8 months; mPFS: 3.1 months vs 1.5 months). Meanwhile, the ORR, time to progression, and disease control rate (DCR) were also been increased. The AEs in the regorafenib group were similar to those in the sorafenib group, including high blood pressure, hand and foot skin reactions, fatigue, and diarrhoea [129]. Encouragingly, the study results of sorafenib-regorafenib sequential therapy demonstrated that from sorafenib treatment to death, the mOS was 26.0 months in the regorafenib group and 19.2 months in the placebo group [130]. There is evidence that the response rate to regorafenib is correlated with HCC molecular subtypes [131]. Therefore, regorafenib can bring significant survival benefits and can be used as an important choice for second-line therapy.

#### Cabozantinib

Cabozantinib is an oral MKI of VEGFR2, MET, AXL, KIT, and RET. In contrast to regorafenib, cabozantinib has been mainly developed as a second-line therapy for Child-Pugh A HCC. A phase 3 study of cabozantinib in the second-line setting for patients with advanced HCC showed improvement in OS, mPFS, and DCR compared with a placebo [132]. Cabozantinib increased the mPFS to 5.2 months from 1.9 months in the placebo group, and the risk of death in the cabozantinib group was reduced by 24%. The ORR was also significantly improved by 4.0% in the cabozantinib group and 0.4% in the placebo group. The majority of AEs were hand-foot syndrome, hypertension, fatigue, and transaminase elevation. The efficacy of cabozantinib is not completely consistent with the liver function of patients, but it shows a trend of a higher curative effect in patients with better liver function [133].

#### Ramucirumab

Briefly, ramucirumab is an anti-VEGFR2 mAb that directly targets the extracellular domain of VEGFR2 and prevents ligand-independent VEGFR2 signalling [134]. Compared with a placebo, ramucirumab did not greatly increase the survival time of unselected HCC patients [135]. However, ramucirumab increased the OS of advanced HCC patients with AFP > 400 ng/ml from 7.3 months to 8.5 months [136]. In addition, from 2015 to 2017, testing was conducted on 292 patients who still had tumour progression after receiving sorafenib. The primary inclusion criteria were impaired liver function, an AFP level of 400 ng/mL or greater, and resistance to sorafenib or progressive disease under sorafenib. The mOS and PFS were significantly increased in this specific group with approximately the same conclusions as previously [137]. Therefore, ramucirumab is a feasible second-line drug in advanced HCC patients with high AFP levels.

#### Nivolumab

Nivolumab is the first anti-PD-1 antibody approved for the treatment of patients with HCC. In the CheckMate040 study, the manageable safety and durable efficacy of nivolumab treatment were indicated in ITT and Asian patients previously treated with sorafenib. The study results show an ORR of 20% and a DCR of 60%, and the efficacy is long lasting [138]. Unfortunately, as a first-line treatment for advanced HCC in CheckMate459, the primary endpoint of the nivolumab group (OS) was not statistically significant compared with sorafenib [68]. Following the results of the CheckMate040 study, nivolumab was conditionally granted approval by the FDA for the second-line treatment of HCC.

#### Pembrolizumab

Pembrolizumab, another antibody against PD-1, is commonly considered a second-line treatment option for HCC. This decision is based on a nonrandomized KEYNOTE-224 study [81]. In this trial, a mOS of 12.9 months, a mPFS of 4.9 months, an ORR of 17%, and a DCR of 61% were recorded. The results of the subsequent randomized phase III trial have also been reported. The study results show that the preset statistically significant endpoint was not reached; however, the ORR of the pembrolizumab group was much higher than that of the placebo group (18.3% vs. 4.4%) [139]. Additionally, Asian people who received pembrolizumab had greater benefits with regard to OS, with an HR of 0.548 (95% CI 0.374, 0.804, *P* = 0.0009), and the survival benefit was better than that of patients in western regions [140].

#### Nivolumab plus ipilimumab

Recently, nivolumab plus ipilimumab was authorized by the FDA as a second-line treatment option for patients with advanced HCC who have received sorafenib. This novel treatment regimen achieved the combination blockade of the immune checkpoints PD-1 and CTLA-4, thereby improving the therapeutic effect for advanced HCC [141]. In the CheckMate-040 study, the efficacy and safety of the combination therapy with nivolumab and ipilimumab were assessed. This combination therapy appears to be safe and tolerable with a confirmed ORR of 33%. As another main efficacy outcome, the duration of response ranged from 4.6 months to 30.5+ months, with 31% of responses lasting at least 24 months. Moreover, combination therapy has shown common AEs similar to those seen with single-drug use. Subsequently, there have been additional clinical studies to evaluate the efficacy of combination immune checkpoint blockade for advanced HCC.

### Challenges and future directions

**Fig. 3:**
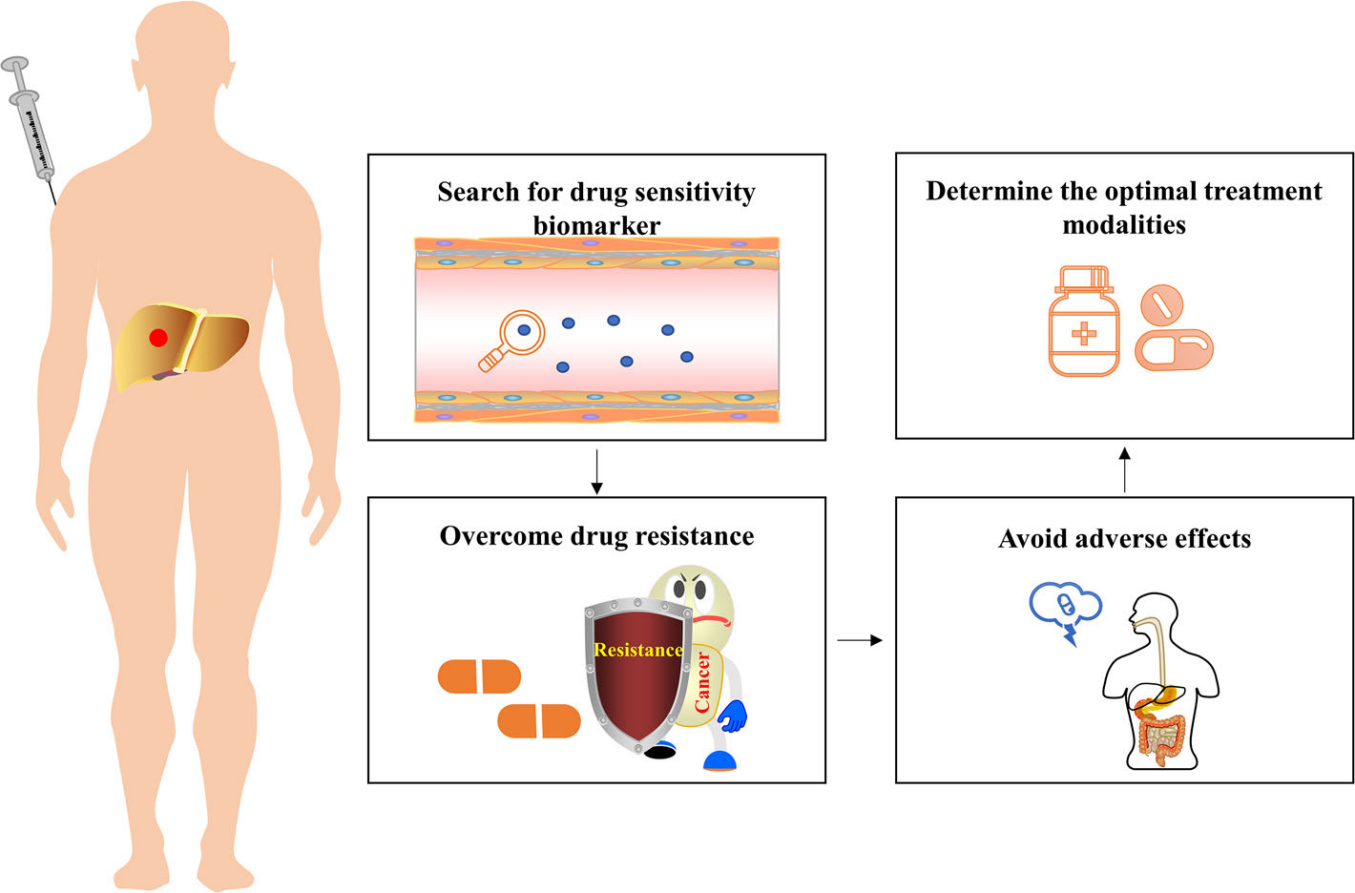
Future directions in targeted therapy for HCC

There are several issues that need to be addressed soon with regard to the treatment of HCC (Fig. 3). Specifically, some biomarkers may be instructive for patient sensitivity to drugs and prognostic implications, such as AFP, which successfully influences treatment decisions in HCC [136]; no additional biomarkers have yet fulfilled this function. While extensive studies have been conducted to examine potential biomarkers to manage HCC [142–145], no available regimen has been developed to treat this tumour. This situation leads to the additional point that the optimal sequence strategy for these new agents is not yet clear. Therefore, it is critical to develop drug sensitivity biomarkers and prognostic biomarkers that identify patients who respond to appropriate treatments, to adopt a novel treatment strategy. Systematic and standardized collection and analysis of blood samples and tumour tissues from patients with HCC should be performed to further develop biomarker-based research.

In the clinic, drug resistance is also a major obstacle to systemic therapy for the treatment of HCC, particularly when newly developed drugs for HCC do not have remarkable results similar to those of other malignancies. The known mechanisms associated with resistance to targeted therapy include epigenetic biological processes, transport processes, regulated cell death, and the tumour microenvironment [146, 147]. Drug resistance, i.e., primary resistance and acquired resistance, is still the main reason for treatment failure during targeted therapy [148]. In addition, the AEs caused by the drugs themselves can have undesirable impacts on the health-related quality of life for patients, even leading to drug discontinuation [149]. These conditions can also be alleviated with combination and sequential therapies.

Finally, it is worth considering how to establish relevant preclinical models based on the heterogeneity of HCC and the tumour microenvironment and then integrate them with data collected from the human body.

## Conclusions

Systemic treatment for patients with HCC is changing due to ever-improving molecular targeted therapies and immunotherapies. The evolution of targeted therapies provides rich data and promising results, particularly in patients who are not amenable to locoregional treatments.

The key success lies in tyrosine kinase inhibitors, which remarkably increase overall survival as a systemic therapy. There is hope for treating HCC at an early or intermediate stage with a better understanding of the molecular mechanisms of hepatocarcinogenesis or the discovery of potential biomarkers. Targeted and immune-based therapies will gradually become the standard treatment for HCC, offering a new strategy for advanced HCC treatment and management. Angiogenesis inhibitors and immune checkpoint inhibitors remain the mainstream approaches to treat HCC. Of these, the research results of several targeted agents in recent years have been successful.

Various clinical trials of systemic therapies are ongoing and have proven successful thus far. These trials are useful in determining the role and effect of molecular targeted therapies and immunotherapies, defining the optimal combinations, and assessing the optimal timing, i.e., adjuvant therapy and first-line or second-line therapy, and thus establishing rational treatment principles. Further exploration of targeted and immunotherapies in HCC is of great practical importance for controlling disease progression, improving the survival quality of patients, and guiding the rational use of clinical drugs.

## Data Availability

Not applicable.

## Abbreviations

AE: adverse event
AFP: alpha fetoprotein
ASCO: American Society of Clinical Oncology
BCLC: Barcelona Clinic Liver Cancer
CI: confidence interval.
CIK: cytokine-induced killer
DCR: disease control rate
EGFR: epidermal growth factor
FDA: Food and Drug Administration
FGF: fibroblast growth factor
HBV: hepatitis B virus
HCC: hepatocellular carcinoma
HCV: hepatitis C virus
HR: hazard ratio.
ICIs: immune checkpoint inhibitors
IGFR: insulin-like growth factor receptor
JAK/STAT: Janus kinase/signal transducer activator of transcription factor pathway
LAG-3: lymphocyte activation gene-3
mAb: monoclonal antibodies
mOS: median overall survival
mPFS: median progression-free survival
ncRNA: noncoding RNA
MKI: multikinase inhibitor
NA: not available
NE: not evaluated
ORR: object response rate
OS: overall survival
PDGF: platelet-derived growth factor
PD-1: programmed death-1
PD-L1: programmed death ligand-1
PFS: progression-free survival
PI3K-AKT-mTOR: phosphoinositide 3-kinase–AKT–mammalian target of rapamycin pathway
PR: partial response
PTEN: phosphate and tension homology deleted on chromosome ten
Ras-Raf-MAPK: mitogen-activated protein kinase pathway
RECIST: response evaluation criteria in solid tumours
RFS: relapse-free survival.
TGF-β: transforming growth factor-β
TIGIT: T cell Ig and immunoreceptor tyrosine-based inhibitory motif domain
TIM-3: T cell immunoglobulin and mucin-containing protein 3
TKI: tyrosine kinase inhibitor
TTP: time to progression
VEGF: vascular endothelial growth factor
VISTA: V-domain Ig suppressor of T cell activation.
